# Sustained release of brimonidine from polydimethylsiloxane-coating silicone rubber implant to reduce intraocular pressure in glaucoma

**DOI:** 10.1093/rb/rbad041

**Published:** 2023-04-24

**Authors:** Chang Huang, Yuening Shen, Yujin Zhao, Zhutian Zhang, Shunxiang Gao, Jiaxu Hong, Jianjiang Xu, Qingtao Meng, Xinghuai Sun, Jianguo Sun

**Affiliations:** Eye Institute and Department of Ophthalmology, Eye & ENT Hospital, Shanghai Medical College, Fudan University, Shanghai 200031, China; NHC Key Laboratory of Myopia, Fudan University, Shanghai 200031, China; Key Laboratory of Myopia, Chinese Academy of Medical Sciences, Shanghai Key Laboratory of Visual Impairment and Restoration, Shanghai 200031, China; Eye Institute and Department of Ophthalmology, Eye & ENT Hospital, Shanghai Medical College, Fudan University, Shanghai 200031, China; NHC Key Laboratory of Myopia, Fudan University, Shanghai 200031, China; Key Laboratory of Myopia, Chinese Academy of Medical Sciences, Shanghai Key Laboratory of Visual Impairment and Restoration, Shanghai 200031, China; Eye Institute and Department of Ophthalmology, Eye & ENT Hospital, Shanghai Medical College, Fudan University, Shanghai 200031, China; NHC Key Laboratory of Myopia, Fudan University, Shanghai 200031, China; Key Laboratory of Myopia, Chinese Academy of Medical Sciences, Shanghai Key Laboratory of Visual Impairment and Restoration, Shanghai 200031, China; Eye Institute and Department of Ophthalmology, Eye & ENT Hospital, Shanghai Medical College, Fudan University, Shanghai 200031, China; NHC Key Laboratory of Myopia, Fudan University, Shanghai 200031, China; Key Laboratory of Myopia, Chinese Academy of Medical Sciences, Shanghai Key Laboratory of Visual Impairment and Restoration, Shanghai 200031, China; Eye Institute and Department of Ophthalmology, Eye & ENT Hospital, Shanghai Medical College, Fudan University, Shanghai 200031, China; NHC Key Laboratory of Myopia, Fudan University, Shanghai 200031, China; Key Laboratory of Myopia, Chinese Academy of Medical Sciences, Shanghai Key Laboratory of Visual Impairment and Restoration, Shanghai 200031, China; Eye Institute and Department of Ophthalmology, Eye & ENT Hospital, Shanghai Medical College, Fudan University, Shanghai 200031, China; NHC Key Laboratory of Myopia, Fudan University, Shanghai 200031, China; Key Laboratory of Myopia, Chinese Academy of Medical Sciences, Shanghai Key Laboratory of Visual Impairment and Restoration, Shanghai 200031, China; Eye Institute and Department of Ophthalmology, Eye & ENT Hospital, Shanghai Medical College, Fudan University, Shanghai 200031, China; NHC Key Laboratory of Myopia, Fudan University, Shanghai 200031, China; Key Laboratory of Myopia, Chinese Academy of Medical Sciences, Shanghai Key Laboratory of Visual Impairment and Restoration, Shanghai 200031, China; School of Chemical Engineering, University of Science and Technology Liaoning, Anshan, Liaoning 114051, China; Eye Institute and Department of Ophthalmology, Eye & ENT Hospital, Shanghai Medical College, Fudan University, Shanghai 200031, China; NHC Key Laboratory of Myopia, Fudan University, Shanghai 200031, China; Key Laboratory of Myopia, Chinese Academy of Medical Sciences, Shanghai Key Laboratory of Visual Impairment and Restoration, Shanghai 200031, China; State Key Laboratory of Medical Neurobiology and MOE Frontiers Center for Brain Science, Institutes of Brain Science, Fudan University, Shanghai 200032, China; Eye Institute and Department of Ophthalmology, Eye & ENT Hospital, Shanghai Medical College, Fudan University, Shanghai 200031, China; NHC Key Laboratory of Myopia, Fudan University, Shanghai 200031, China; Key Laboratory of Myopia, Chinese Academy of Medical Sciences, Shanghai Key Laboratory of Visual Impairment and Restoration, Shanghai 200031, China; State Key Laboratory of Medical Neurobiology and MOE Frontiers Center for Brain Science, Institutes of Brain Science, Fudan University, Shanghai 200032, China

**Keywords:** sustained release, conjunctival sac implant, glaucoma, intraocular pressure, brimonidine

## Abstract

Glaucoma is the leading cause of irreversible blindness, affecting 111 million people by 2040 worldwide. Intraocular pressure (IOP) is the only controllable risk factor for the disease and current treatment options seek to reduce IOP via daily taking eye drops. However, shortcomings of eye drops, such as poor bioavailability and unsatisfied therapeutic effects, may lead to inadequate patient compliance. In this study, an effective brimonidine (BRI)-loaded silicone rubber (SR) implant coated with polydimethylsiloxane (BRI@SR@PDMS) is designed and fully investigated for IOP reduction treatment. The *in vitro* BRI release from BRI@SR@PDMS implant reveals a more sustainable trend lasting over 1 month, with a gradually declined immediate drug concentration. The carrier materials show no cytotoxicity on human corneal epithelial cells and mice corneal epithelial cells *in vitro*. After administrated into rabbit’s conjunctival sac, the BRI@SR@PDMS implant releases BRI in a sustained fashion and effectively reduces IOP for 18 days with great biosafety. In contrast, BRI eye drops only maintain IOP-lowering effect for 6 h. Therefore, as a substitute of eye drops, the BRI@SR@PDMS implant can be applied as a promising non-invasive platform to achieve long-term IOP-lowering in patients suffering from ocular hypertension or glaucoma.

## Graphical Abstract

**Figure:**
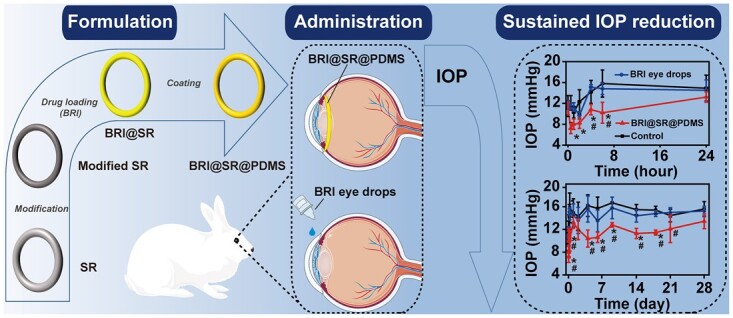

## Introduction

Globally, glaucoma is the leading cause of irreversible vision impairment and blindness, and there will be 111 million patients by 2040 [1]. Glaucoma is a group of neurodegenerative disorders characterized by elevated intraocular pressure (IOP) as well as progressive and irreversible retinal ganglion cells loss [2], and the only modifiable risk factor for glaucoma progression is the elevated IOP [3]. Currently, several agents such as pilocarpine, α-adrenergic agonists, β-blockers, prostaglandin analogs, carbonic anhydrase inhibitors and Rho-kinase inhibitor have been used to reduce IOP in glaucoma patients [4]. Among them, brimonidine (BRI) is a kind of α_2_-selective adrenergic agonists which can lower IOP via reducing aqueous humor production and stimulating uveoscleral outflow [5]. However, only 1–7% of eye drops administrated on ocular surface can reach into eye tissues (low bioavailability) due to tear turnover, nasolacrimal drainage, reflex blinking and corneal barriers [6, 7]. IOP-lowering effects of eye drops last only a few hours, and thus multiple administrations (2–3 per day) are required. Furthermore, glaucoma is a chronic disease and a lifelong treatment is needed. Eye drops are simple, convenient and widely used, but they have low bioavailability and much unavailable drugs may cause drug-induced tissue toxicity after long-term administration [8]. These factors finally lead to inadequate patient compliance, and only 31–67% patients can comply to their treatment regimens for a year [9]. Therefore, it is quite vital to seek for alternative formulations with improved drug bioavailability and therapeutic effectiveness for anti-glaucoma therapy [10].

Much efforts have been focused on developing new types of formulations for glaucoma treatment, such as drug delivery systems [11] based on microspheres [12], nanoparticles [13–15], *in situ* gels [16], dendrimers [17], niosomes [18], micelles [19], liposomes [20], contact lens [21] and conjunctival sac implants [22]. Among the conjunctival sac implants, silicone rubber (SR) implants have attracted a lot of attention due to their desirable properties including adjustable flexibility and stiffness [23], inertia as well as biocompatibility [24], and they possess great capacity to develop drug delivery systems [25], ocular implants [26] and scleral buckling [27]. Our previous study [28] also reported a BRI-loaded SR implant and described its effectiveness for BRI sustainable-release and IOP-lowering. However, the sample preparation stability due to insufficient attachment of thermoplastic polyurethane coating on the SR implants hindered the further development toward a commercial formulation. As we all known, polydimethylsiloxane (PDMS), also named as SR, has been widely used in medical fields such as islet transplantation [29], microfluidic device [30], skin patch [31], contact lens [32], glaucoma drainage device [33–35] and surface coating [36]. Thus, it can be predicted that PDMS coating should have a perfect attachment capacity on SR surface due to the same molecular structures.

**Scheme 1.**
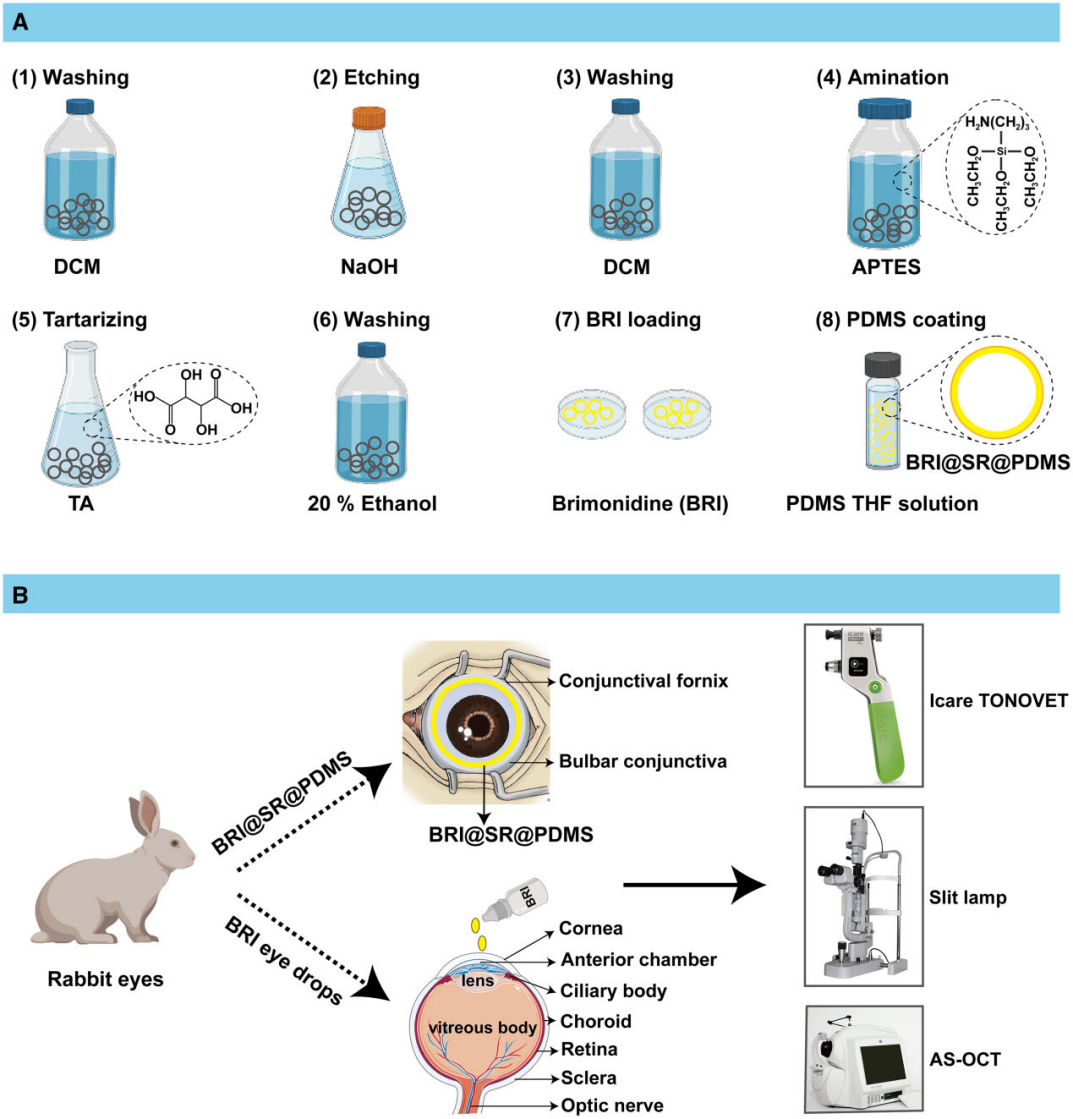
Schematic diagram of (A) preparation process of BRI@SR@PDMS implant and (B) administration of BRI@SR@PDMS *in vivo*.

In this study, we aimed to incorporate BRI into PDMS-coated SR to develop a new BRI@SR@PDMS implant for IOP-lowering treatments (as shown in Scheme 1). We investigated the properties and cytotoxicity of BRI@SR@PDMS implants as well as the BRI release profile *in vitro*. The *in vivo* biosafety as well as treatment effectiveness of the conjunctival sac-administrated BRI@SR@PDMS implant were further evaluated in rabbit eyes.

## Materials and methods

### Materials

BRI was purchased from J&K Scientific Co., Ltd (Shanghai, China). SR was supplied by Kangda Rubber Co., Ltd (Shanghai). PDMS was obtained from Ausbond Co., Ltd (Shenzhen, China). Tartaric acid (TA), 1-(3-dimethylaminopropyl)-3-ethylcarbodiimide hydrochloride (EDC), *N*-hydroxysuccinimide (NHS) and Dulbecco’s Modified Eagle Media (DMEM/F12) were obtained from Sigma-Aldrich (Shanghai). Aminopropyl triethoxy silane (APTES) was supplied by Aladdin (Shanghai). Sodium hydroxide (NaOH), tetrahydrofuran, anhydrous ethanol and dichloromethane (DCM) were obtained from Lingfeng Co., Ltd (Shanghai). Cell Counting Kit-8 (CCK-8) assay was bought from Dojindo Molecular Technologies, Inc. (Shanghai). Calcein acetoxymethyl ester (Calcein-AM) and propidium iodide (PI) were obtained from Servicebio Co., Ltd. (Wuhan, China). PriMed-iCELL-001 was purchased from Ruian BioTechnologies Co., Ltd (Shanghai). Fetal bovine serum (FBS) and phosphate-buffered saline (PBS) were obtained from Thermo Fisher Scientific (Shanghai). Besides, xylazine hydrochloride (Jilin TAT, Jilin, China), 0.15% BRI tartrate eye drops (Allergan, TX, USA) and oxybuprocaine hydrochloride eye drops (Santen, Shanghai) were purchased and used as received. All other chemicals and reagents were of analytical grade.

### Preparation and characterization of BRI@SR@PDMS implant

#### Surface modification of SRs

Surface modifications of SRs were performed according to the reported methods [28]. In a typical process, the SRs (*n* = 40) were immersed in 10 ml DCM for 10 min and then dried naturally. After the above washing courses were repeated thrice, the SRs were dried under vacuum for 1 h. Subsequently, those SRs were placed into NaOH-ethanol aqueous solution (80 ml, 4 M, 50% H_2_O, 80°C) and stirred at a speed of 100 rpm for 4 h to loosen the internal microstructure of the SRs [37], followed by washing with ethanol aqueous solution (50 ml, 50%) and DCM (10 ml). After 12 h-vacuum drying, the etched SRs were soaked in the APTES ethanol solution (100 ml, 0.1%, 70°C, 80 rpm) for 0.5 h, washed with ethanol (100 ml) for 0.5 h, dried at 110°C for 3 h and eventually vacuumized for 24 h. The aminated SRs were then soaked in TA ethanol aqueous solution (100 ml, 0.4 mg/ml, 50% H_2_O, pH = 5.5, 25°C) with EDC (960 mg) and NHS (590 mg) for 24 h. After these procedures, the TA-modified SRs were then washed 3 times with 20% ethanol aqueous solution for 30 min each, and dried at 37°C for 12 h. Finally, the surface-modified SRs were obtained.

#### Preparation of BRI@SR@PDMS implant

After 400 mg BRI was dissolved in 40 ml DCM with 10 ml methyl alcohol, the surface-modified SRs were added into the above BRI solution and fully swelled within 30 min, followed by drying naturally in a fume hood. Then, the BRI-loaded SRs were swelled in the above BRI solution for 5 min again and drying naturally, and the drug loading course was repeated thrice. Without any cleaning steps, the BRI-loaded SRs were further impregnated into 10 ml THF solution with PDMS precursor (10% w/w) for 5 s, followed by slowly volatilizing and drying naturally in a fume hood to form PDMS coating on the surface of the BRI-loaded SRs. The above coating course was repeated for 2, 4 or 8 times. BRI@SR@PDMS implants with different PDMS coating times were identified as BRI@SR@PDMS (10% × 2), BRI@SR@PDMS (10% × 4) and BRI@SR@PDMS (10% × 8).

#### Characterization of BRI@SR@PDMS implant

The surface morphology of SRs (unmodified), modified SRs, BRI@SRs and BRI@SR@PDMS implants was observed and the images were captured by a digital camera. Their interfacial morphology was observed with a scanning electron microscopy (SEM, Zeiss Sigma 300, Oberkochen, Germany), after a 0.5-mm-thick cross-section of SR samples was obtained and coated with a 10-nm gold layer. Surface modifications of the SRs and BRI-loading into the SRs were confirmed using Fourier transform infrared spectroscopy (FTIR) spectrophotometer (Model 22, Bruker, Coventry, UK) with scanning range of 600–4000 cm^−1^ in an attenuated total reflection mode as previously reported [38].

Residual volatile substance and thermal stability of these four kinds of samples were evaluated by thermogravimetric and differential scanning calorimeter (TG-DSC, Perkin Elmer, Waltham, MA, USA) according to the reported method [39]. Tensile measurements were carried out on a universal testing machine (Instron Ernst Brinck, Canton, MA, USA) at a rate of 500 mm/min (25°C) with 15-mm extensometer grips and all samples were tested under same conditions.

### Sustained release of BRI from BRI@SR@PDMS implant

The *in vitro* BRI release profile from BRI@SR@PDMS implant was investigated using a previously reported method [28]. Three kinds of the BRI-loaded samples containing the same amount of BRI, including BRI@SR@PDMS (10% × 2), BRI@SR@PDMS (10% × 4) and BRI@SR@PDMS (10% × 8), were investigated in the study. The sample was put into a 50 ml-centrifuge tube and PBS (10 ml) was then added as the release medium. The centrifuge tubes with different samples were placed in the DKZ-3B shaker (Yiheng, Shanghai, China) with a stirring speed of 50 rpm and temperature of 37°C. At each set time point (0, 1, 4, 8 h, and 1, 2, 4, 8, 14, 21, 28 and 35 days), 2 ml of the medium was collected and another 2 ml of pre-warmed fresh PBS was then added to the release medium. Concentrations of BRI in the collected samples were determined at 246 nm using a UV–Vis spectrophotometer (NanoDrop 2000, Thermo Scientific, USA). For each kind of samples, the result was obtained by calculating the mean of four replicates (*n* = 4). The release profile of BRI from the BRI-loaded samples was analyzed according to the measured results.

### *In vitro* cytotoxicity of BRI@SR@PDMS implant

To evaluate the cytotoxicity of BRI@SR@PDMS implants *in vitro*, the survival and viability of human corneal epithelial cells (HCECs) and mice corneal epithelial cells (MIC-iCELL-m001) were investigated after coculturing them with the leaching liquor of BRI@SR@PDMS implants, BRI@SRs or SRs. HCECs were purchased from the American Type Culture Collection (ATCC, Manassas, VA), and MIC-iCELL-m001 was bought from Ruian BioTechnologies Ltd. Co. (Shanghai). MIC-iCELL-m001 was cultured in PriMed-iCELL-001 containing 10% FBS. The leaching liquor of BRI@SR@PDMS implants, BRI@SRs or SRs was prepared by incubating the sterilized samples with culture medium for 24 h at 37°C according to the International Standard for Biological Testing of Medical Devices (0.2 g/ml culture medium) [40].

Cells were cultivated at a density of 1.0 × 10^4^ cells/well in 96-well plates (*n* = 10) with the leaching liquor of BRI@SR@PDMS implants, BRI@SRs or SRs for 24 h. The live and dead cells were labeled via Calcein-AM/PI staining according to the reported method [41]. The cells were washed with pre-warmed PBS twice, after which they were shielded and incubated with Calcein-AM (10 μM, 50 μl/96-well, *n* = 6) for 0.5 h. Then the cells were washed with PBS twice again, and stained with PI (5 μM, 50 μl/96-well, *n* = 6) for 10 min. Finally, the cells were observed using fluorescence microscopy (Leica, Germany). Furthermore, cell viability was detected by CCK-8 assay as previously reported methods [28]. After the medium was removed, CCK-8 solution diluted with respective culture medium was added to 96-well and incubated for another 2 h (37°C, 5% CO_2_). The UV absorbance of each well was detected by a micro plate reader (Synergy H1 Hybrid Reader, BioTek, Winooski, VT, USA) at 450 nm. The cells cultured in a normal medium were used as the control, and their viability was set as 100%. The cytotoxicity of BRI@SR@PDMS implants, BRI@SRs or SRs (*n* = 4) *in vitro* was shown as the relative percentage of cell viability.

### IOP-lowering effectiveness and biosafety of BRI@SR@PDMS implant

The study was conducted according to the guidelines of the Animal Care and Use Committee of Fudan University (Shanghai), under reference No. IACUC-DWZX-2021-022. All rabbit experimental protocols, including transportation, care and operations, complied with the Association for Research in Vision and Ophthalmology Statement for the Use of Animals in Ophthalmic and Vision Research. The animals were housed under standard conditions in the facility of the Eye & ENT Hospital (Fudan University), with free access to food, water and fresh air throughout the experimental period and temperature was maintained at 25°C with relative humidity of 60 ± 10% and 12 h alternate light and dark cycle.

New Zealand rabbits (male, ∼2 kg) were supplied by Shanghai Bikai Keyi Biotechnology Co., Ltd (Shanghai China) and housed for 2 weeks for environmental adaptation. The right eyes of 10 rabbits were randomized into either BRI@SR@PDMS implant group (*n* = 5) or BRI eye drop group (*n* = 5), and all left untreated eyes served as the blank control (*n* = 10). Animal anesthesia was achieved by an intramuscular injection of xylazine hydrochloride (10 mg/kg) and topical application of 0.5% oxybuprocaine hydrochloride eye drops. In the BRI@SR@PDMS implant group, one BRI@SR@PDMS implant was administrated into the rabbit conjunctival sac, followed by a stitch (Lambert’s stitching) to prevent possible scratching-off. In the BRI eye drop group, BRI eye drops were applied on the corneal surface (18 µl, 5 times, and 10 min interval for each dose). The anterior segments of all rabbit eyes were observed using optical coherence tomography (OCT) and a slit lamp. The representative photographs were taken by a digital camera. The corneal thickness in all groups was measured by ImageJ, based on the OCT images of the rabbit eye anterior segments.

#### In vivo drug release

After the administration of BRI@SR@PDMS implant or BRI eye drops (0.15%) to rabbit eyes (*n* = 5), 100 μl of aqueous humor was extracted with a 1-ml syringe at 1, 6, 24, 48, 96 and 192 h. All aqueous humor samples were stored at −80°C before the analysis with liquid chromatography-mass spectrometry system as previous described [42].

#### IOP measurement

IOP was measured using an Icare^®^ TONOVET Plus rebound tonometer (Icare Finland Oy, Helsinki, Finland) at 0, 0.5, 1, 2, 4, 6 h, and 1, 2, 4, 6, 9, 14, 18, 21 and 28 days after the administration of BRI@SR@PDMS implant or BRI eye drops. In order to decrease possible discomfort caused by tonometer contact, rabbit eyes were topically anesthetized by oxybuprocaine hydrochloride eye drops before IOP measurements.

#### Inflammatory cytokines

Aqueous humor (100 μl) was collected from each rabbit eye at the terminal of the whole experiment (Day 28). The concentrations of inflammatory cytokines (Interleukin-1β (IL-1β) and Tumor Necrosis Factor-alpha (TNF-α)) were measured by enzyme-linked immunosorbent assay Kits (Muti Sciences, Beijing, China) according to the manufacturer’s detailed instructions.

#### Histological analysis

After the 28-days observation, all rabbits were sacrificed by overdose of anesthetics. The eyeballs were then rapidly removed, fixed in pre-chilled 4% paraformaldehyde and embedded in paraffin. The eyeballs were then sectioned (3 µm) and stained with hematoxylin & eosin (H&E) and Masson. The sections were observed by Leica optical microscope (Wetzlar, Germany).

### Statistical analysis

Each experiment was conducted at least 3 times independently and data were shown as mean ± SD. One-way ANOVA with SPSS (SPSS Inc., Chicago, IL, USA) was performed for statistical analysis. The results were considered statistically significant for *P *<* *0.05.

## Results and discussions

### Characterization of BRI@SR@PDMS implant

#### Morphology analysis of BRI@SR@PDMS implant

**Figure 1.**
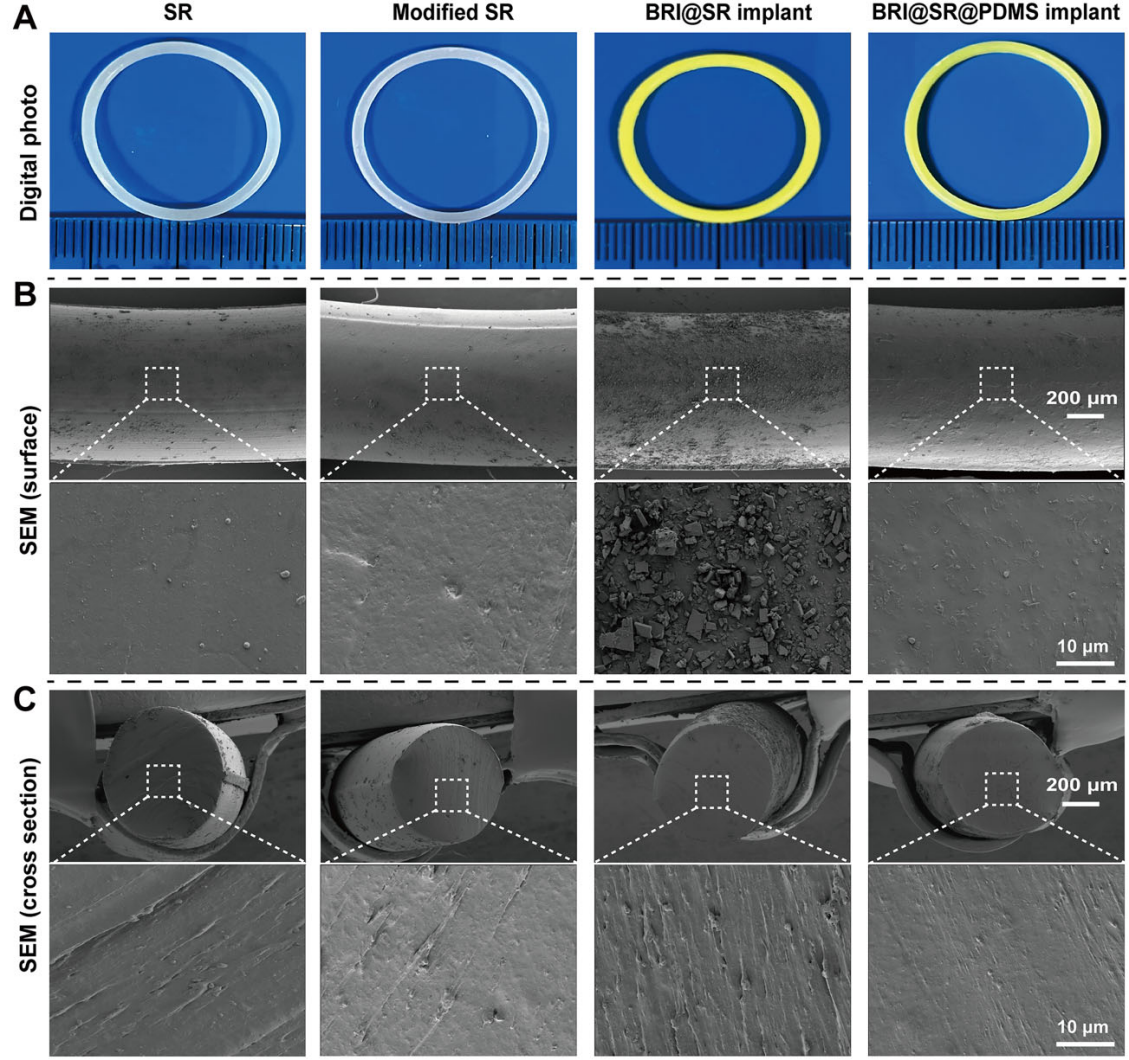
Surface and interfacial morphology images of SRs with and without surface modifications, BRI-loading and PDMS-coating. (A) Representative photographs of the samples obtained by a digital camera; SEM images of (B) the outer surface and (C) the cross-section of the samples.

The surface and interfacial morphology of the SRs (unmodified), modified SRs, BRI@SRs and BRI@SR@PDMS implants was observed, and the representative images are shown in Fig. 1. The SRs (unmodified) showed smooth surface with a cross-section diameter of 1376.8 ± 37.9 μm (Column 1, Fig. 1). After a series of modifications, the modified SRs showed rough surface with a decreased cross-section diameter of 1234.7 ± 41.8 μm (Column 2, Fig. 1). Subsequently, plenty of granular materials were observed distributing on the surface of BRI-loaded SR samples (Column 3, Fig. 1), which should be attributed to the BRI crystallization after organic solvent volatilization. After the drug-loading course, the BRI-loaded SR samples provided an increased cross-section diameter of 1250.8 ± 22.2 μm. Furthermore, the above granular and coarsened surface of the BRI@SR implants became flattened again after PDMS coating was performed, and their cross-section diameter increased continuously to 1357.2 ± 45.1 μm (Column 4, Fig. 1).

#### FTIR analysis of BRI@SR@PDMS implant

**Figure 2.**
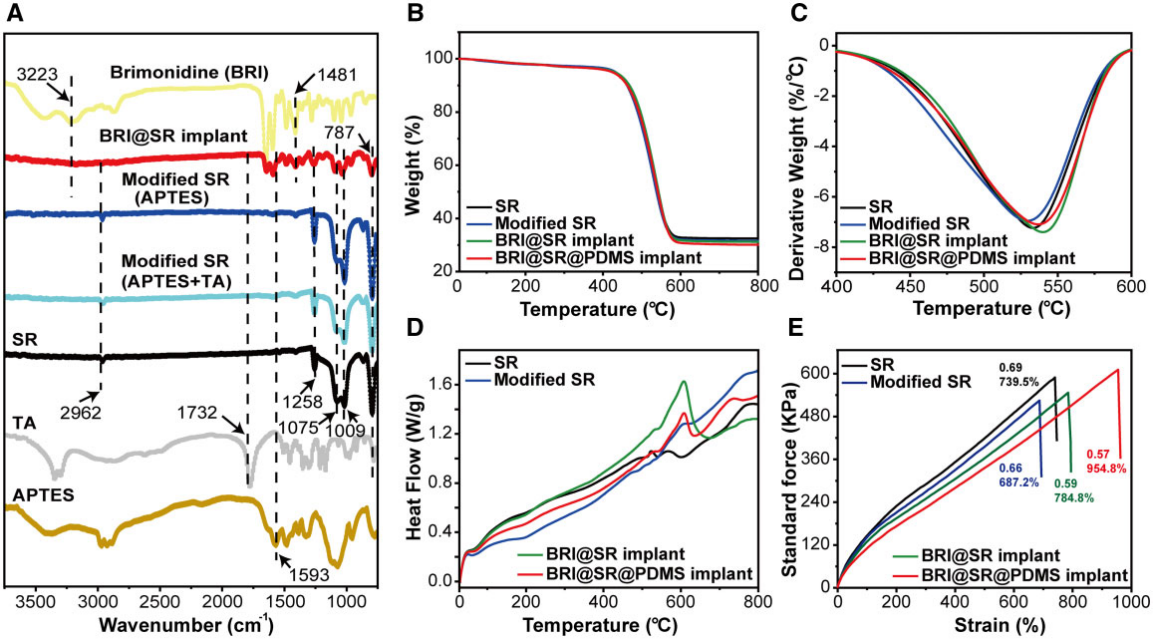
FTIR spectra, thermal stability and tensile strength of BRI@SR@PDMS implants. (A) FTIR spectra of APTES, TA, BRI, the SR samples with and without surface modifications (APTES + TA) and the modified SR samples with BRI-loading; (B) TG and (C) DTG analyses; (D) DSC and (E) tensile stress–strain profiles of the SRs with and without surface-modifications, BRI-loading and PDMS-coating.

In Fig. 2A, the FTIR spectrum of the unmodified SRs (black line) peaked at 2962 cm^−1^ (*v*(C–H)), 1258 cm^−1^ (*δ*(C–H)), 1075 cm^−1^ and 1009 cm^−1^ (δ(Si–O–Si)) and 787 cm^−1^ (Si–CH_3_ wagging), respectively [43]. After APTES and TA modifications, the SRs (light blue line) showed characteristic peaks of APTES (1593 cm^−1^ to *δ* (N–H)) [44] and TA (1732 cm^−1^ to *v*(C=O)) [45], which confirmed that the surface modifications were successfully performed. Similarly, when BRI was loaded into the modified SRs, BRI@SR implants (red line) presented characteristic peaks of BRI (3223 cm^−1^ (*v*(N–H)) and 1481 cm^−1^ (*v*(C–C))) [46], which confirmed the successfully loading of BRI [47]. In order to improve BRI-loading into the SRs, we selected TA to modify the internal microstructure of the SRs due to the appropriate molecular interaction between BRI and TA (brimonidine tartrate) [48]. In fact, the commercial BRI eye drops are made of brimonidine tartrate (0.15%, w/w) [49].

#### Thermal stability and tensile strength of BRI@SR@PDMS implant

Thermal stability before and after series of surface modifications, BRI-loading and PDMS-coating was investigated in the temperature range of 0–800°C, and the results are presented in Fig. 2B. A similar thermal degradation pattern was observed in the TG curves of the samples, and only a little bit difference was found. The difference could be obviously displayed in the derivative thermogravimetric (DTG) curves (Fig. 2C). The DTG curves displayed similar and strong peaks, representing the maximum weight loss change rate and the main pyrolysis stage of the SRs, but they presented different peak positions. The peak of derivative weight change of the unmodified SRs was at 533.4°C, while that of the modified SRs slightly decrease to 530.1°C, which was mainly due to their slightly loose microstructures after the modifications with NaOH, APTES and TA (Fig. 2C). In term of the BRI-loaded SRs (BRI@SR), its peak moved up to 541.1°C. This phenomenon revealed the slightly enhanced internal microstructure, which was mainly due to the drug loading into the internal micropores and the possible molecule interaction between BRI and the grafted TA. After coated by PDMS, the implant (BRI@SR@PDMS) showed a little bit moving-down peak at 537.2°C, which mainly resulted from the raised polymer component. Furthermore, the DSC curves of the SR samples are shown in Fig. 2D, and a mild endothermic peak was proposed around 530.2°C, which was correspondent with the results of DTG. Notably, the DSC curves of BRI@SR and BRI@SR@PDMS experienced an endothermic peak at 608.1°C, which represented the melting course of BRI [46]. It was very interesting that the characteristic peak of BRI observed in the DSC curve of BRI@SR significantly disappeared in that of the PDMS-coated BRI@SR (BRI@SR@PDMS). This was mainly due to the partial inhibition of BRI crystallinity by the PDMS-coating. On the whole, under the biological temperature (37°C), all SR samples exhibited great thermal stability.

The stress–strain curves of the SR samples before and after surface modifications showed a linear change (Fig. 2E), which indicated that they were perfectly elastic. The SRs presented a curve slope of 0.69 and strain of 739.5%, while the modified SRs showed a decreased curve slope (0.66) and strain (687.2%), indicating that the modified SRs were softer and had a looser microstructure. After BRI-loading, the modified SRs (BRI@SR) showed a further decreased slope (0.59) and more strain (784.8%). After PDMS-coating, the BRI@SR@PDMS implant obtained the least slope (0.57) and the most strain (954.8%). That was, the BRI@SR@PDMS implant presented a perfect elasticity and was suitable for further administration *in vivo*.

### *In vitro* drug release from BRI@SR@PDMS implant

**Figure 3.**
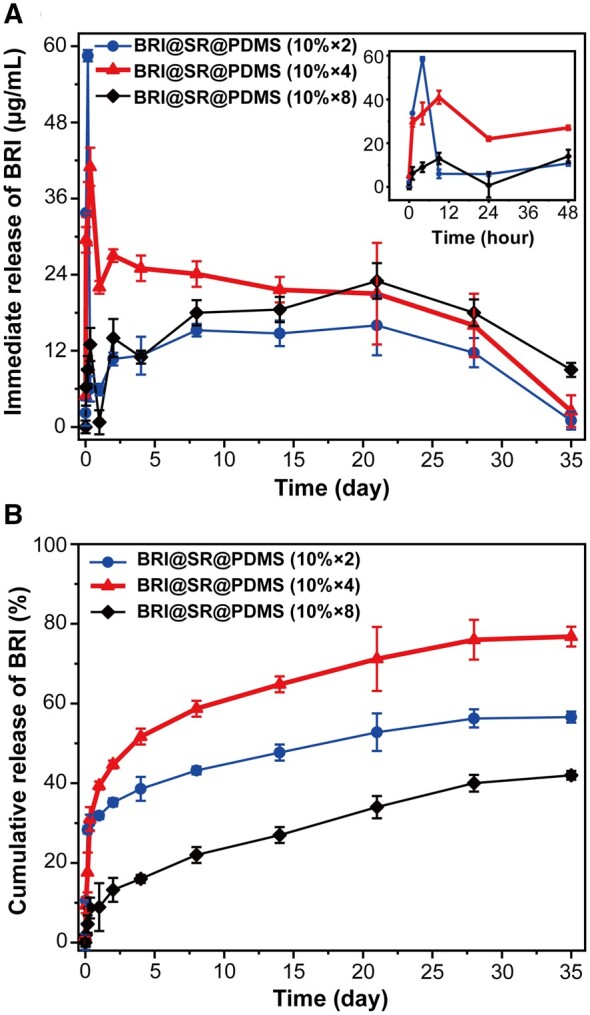
*In vitro* (A) immediate release curves and (B) cumulative release curves of BRI from BRI@SR@PDMS implant (10% × 2), BRI@SR@PDMS implant (10% × 4) and BRI@SR@PDMS implant (10% × 8) (*n* = 4).

In this study, an average amount of 1212.1 ± 37.7 μg BRI was loaded into each BRI@SR@PDMS implant, which was more than double of the average drug-loading amount in the BRI@SR (unmodified) implant (595.8 ± 8.5 μg). The improved drug-loading results verified the necessity and effectiveness of the loose and surface modification of the internal microstructure of the SRs. As shown in Fig. 3A, the BRI@SR@PDMS implant (10%×2) presented the worst ‘burst release’ (*C*_max_ = 58.5 μg/ml) within 4 h, and then the lowest immediate concentration of BRI (<16.3 μg/ml) until 35 days. Increasing the number of PDMS-coating from 2 to 4 significantly ameliorated the ‘burst release’ with the *C*_max_ of 44.3 μg/ml at 8 h, and improved the immediate concentration of BRI (>20.7 μg/ml) until 14 days. Further increasing the number of PDMS-coating to 8 markedly inhibited the ‘burst release’ with the *C*_max_ of 13.2 μg/ml at 8 h, and presented a lower immediate concentration of BRI (<18.5 μg/ml) until 14 days. More importantly, in Fig. 3B, the cumulative release profile of the BRI@SR@PDMS implant (10% × 4) revealed the less ‘burst release’ (17.6%), more sustainable and complete BRI release (∼76.8% at 35 days) than those of the BRI@SR@PDMS implant (10% × 2) (∼28.3% and 56.6% at 35 days) and BRI@SR@PDMS implant (10% × 8) (∼4.6% and ∼42.9% at 35 days). With the coating time of PDMS increasing from 2, 4 to 8, although the ‘burst release’ of BRI from BRI@SR@PDMS implant was significantly improved, the normal BRI release (within 14 days) and the complete BRI release were accidentally inhibited. Thereby, the BRI@SR@PDMS implant (10% × 4) was chosen as the optimized formulation for further investigations, due to its controlled burst release and improved BRI release pattern.

### *In vitro* cytotoxicity of BRI@SR@PDMS implant

**Figure 4.**
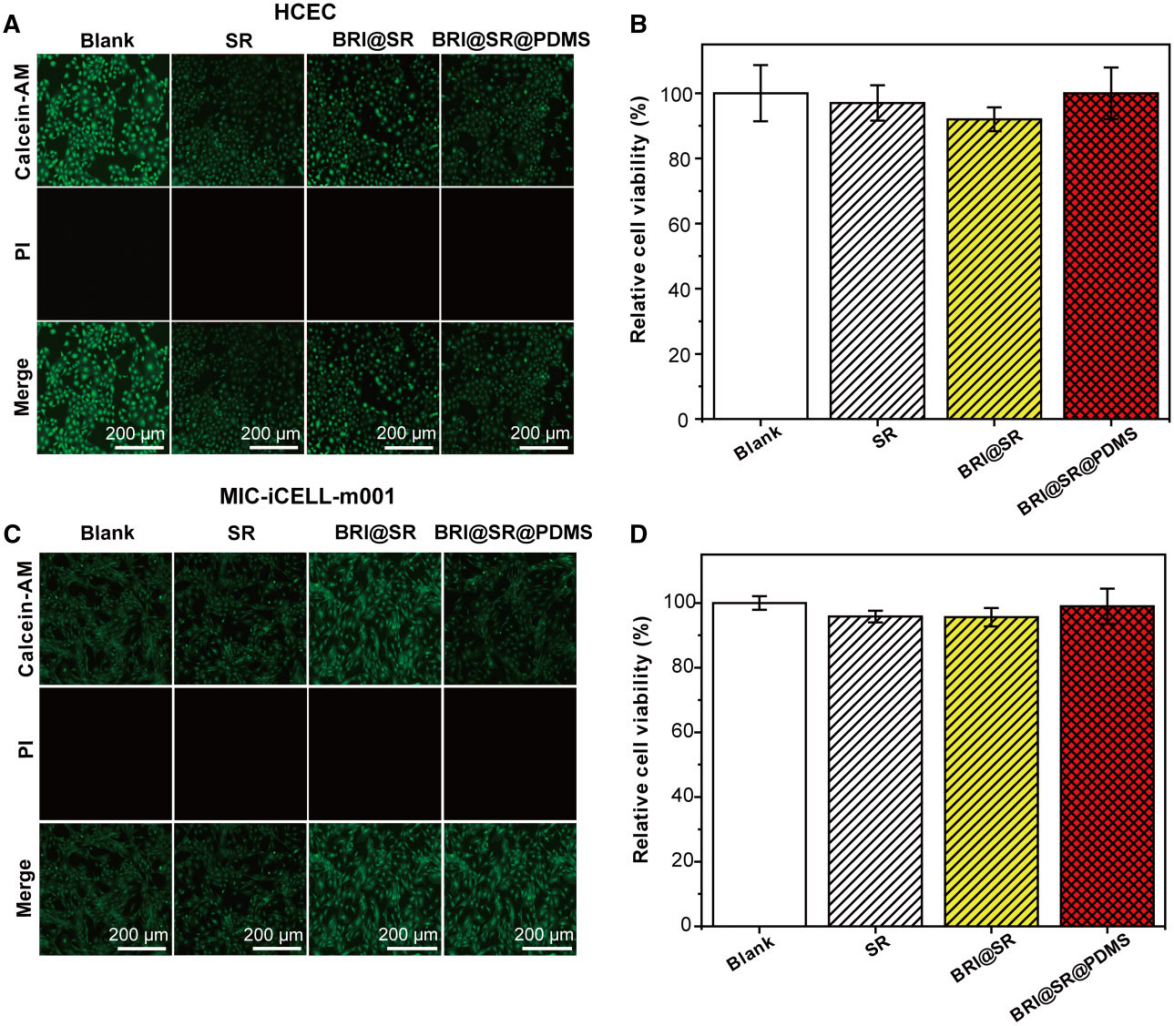
*In vitro* cytotoxicity evaluations of BRI@SR@PDMS implant, BRI@SR and SR. (A) The representative photographs and (B) the statistical analysis of cell viability of HCECs. (C) The representative photographs and (D) the statistical analysis of cell viability of mice corneal epithelial cells (MIC-iCELL-m001) after co-culturing with the leaching liquor of the implants (*n* = 4). The viability of the cells without any manipulation was set as the blank (100%).

The *in vitro* cytotoxicity of BRI@SR@PDMS implant was evaluated by culturing HCEC or MIC-iCELL-m001 using the implant’s leaching liquor. As shown in Fig. 4A, no death cells were observed in the BRI@SR@PDMS implant, BRI@SR and SR groups, similar to the result in the blank group. Moreover, the cell viability results indicated that BRI@SR@PDMS implant, BRI@SR and SR had no obvious toxicity to HCEC (Fig. 4B), and no statistically significant difference was established among BRI@SR@PDMS implant, BRI@SR, SR and the blank (*P *>* *0.05). Moreover, MIC-iCELL-m001 was also used to confirm the *in vitro* safety of BRI@SR@PDMS implant, and the similar results were obtained (Fig. 4C and D). Consequently, BRI@SR@PDMS implant was non-toxic and biocompatible *in vitro*, and they could further be employed for *in vivo* administration and treatment.

### IOP-lowering effectiveness of BRI@SR@PDMS implant

#### In vivo drug release

**Figure 5.**
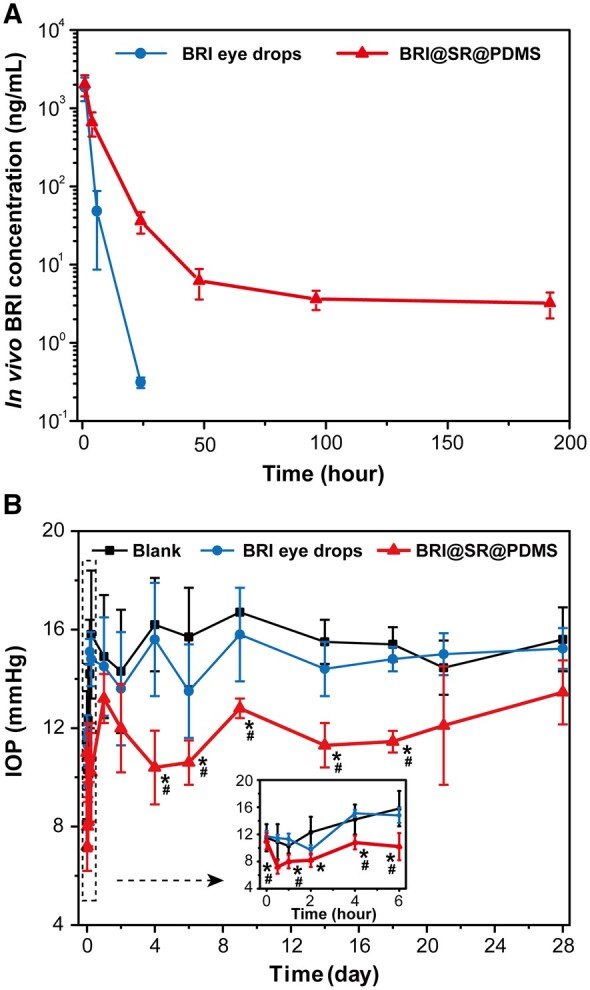
(A) *In vivo* concentration–time curves of BRI in rabbit aqueous humor and (B) IOP curves after topical installation of BRI eye drops (0.15%) or conjunctival sac administration of BRI@SR@PDMS implant to the rabbit right eyes (*n* = 5). All left eyes without any manipulation were set as the blank (*n* = 10). * and ^#^ indicate the statistically significant difference (*P *<* *0.05) compared with the blank and BRI eye drops at each time point, respectively.

The *in vivo* BRI release was studied by measuring the concentration of BRI in rabbit aqueous humor after topical installation of BRI eye drops (0.15%) or conjunctival sac administration of BRI@SR@PDMS implant. As shown in Fig. 5A, the amount of BRI in the anterior chamber reached its highest concentration (1844.0 ng/ml) within 1 h after BRI eye drops application, and then it rapidly decreased to 0.3 ng/ml at 24 h. On the contrary, BRI concentration after administration of BRI@SR@PDMS implant reached to 2028.0 ng/ml within 1 h and 659.7 ng/ml at 6 h, and then it maintained a gradually decline trend with 35.9 ng/ml at 24 h, and 3.2 ng/ml at 192 h. Obviously, BRI@SR@PDMS implant provided a more sustained drug release pattern than BRI eye drops, which proved the benefit in maintaining a long-term and considerable IOP reducing activity.

In this study, a BRI@SR@PDMS implant was administrated into a rabbit conjunctival sac, while 90 µl (18 µl, 5 times and 10 min interval) of BRI eye drops (0.15% w/w) were administrated on the rabbit cornea according to the clinically used dose [50]. The total BRI amount in the BRI@SR@PDMS implant (1212.1 μg) was almost same with that in the BRI eye drops (1350 μg). As we all known, topical administration by dropping conventional eye drops has a very low bioavailability, and the vast majority of drugs administrated on the cornea surface are wasted due to the limited tear volume, conjunctival sac volume [51, 52], and rapid clearance based on blinking and non-target tissue absorption [53]. Thus, the amount of BRI absorbed by rabbit cornea and traveling into anterior chamber is quite limited. On the contrary, the administration of BRI@SR@PDMS implant into rabbit conjunctival sac did not increase tear volume and cause rapid drug clearance based on blinking. The administrated BRI@SR@PDMS implant was immersed in tears and sustainably released BRI to the tears without significant change of tear volume and drug waste.

#### IOP-lowering effectiveness

The therapeutic effectiveness of BRI@SR@PDMS implant was evaluated by measuring IOP once at the set time points after the implant administration. IOP curves are shown in Fig. 5B. The IOP of rabbits without any treatment (the blank group) ranged from 14.2 to 16.7 mmHg and no significant IOP-lowering effect was observed during the study period. In contrast, the IOP in both BRI eye drop group (9.7–11.7 mmHg) and BRI@SR@PDMS implant group (10.6–12.1 mmHg) significantly decreased within 1–2 h after the treatments. Subsequently, the IOP rapidly recovered after 4 h and eventually reached a plateau in the BRI eye drop group, similar to that in the blank group (Fig. 5B). However, the BRI@SR@PDMS implant dramatically lowered IOP and maintained the IOP-lowering effect till Day 18. BRI eye drops failed to maintain IOP-lowering effect after 6 h, which was supported by the rapidly decaying BRI concentration in the aqueous humor (Fig. 5A). These demonstrated that the BRI@SR@PDMS implant successfully reduced IOP in a sustained style compared with the conventional BRI eye drops (18 days versus 6 h, 72 times), confirming the superiority of the implant in the treatment of glaucoma. IOP may be affect by several factors, such as anesthetic sensitivity, measurement frequency, measurement methods, emotional factors and eye trauma [54, 55]. An IOP abnormal fluctuation was observed at Day 2 in the BRI@SR@PDMS implant group, which mainly resulted from the gradually declining anesthetic sensitivity within 24 h and the possible emotional tension induced by the foreign body sensation provided by the BRI@SR@PDMS implant and the small eye trauma caused by the Lambert’s stitching.

### Biosafety of BRI@SR@PDMS implant

**Figure 6.**
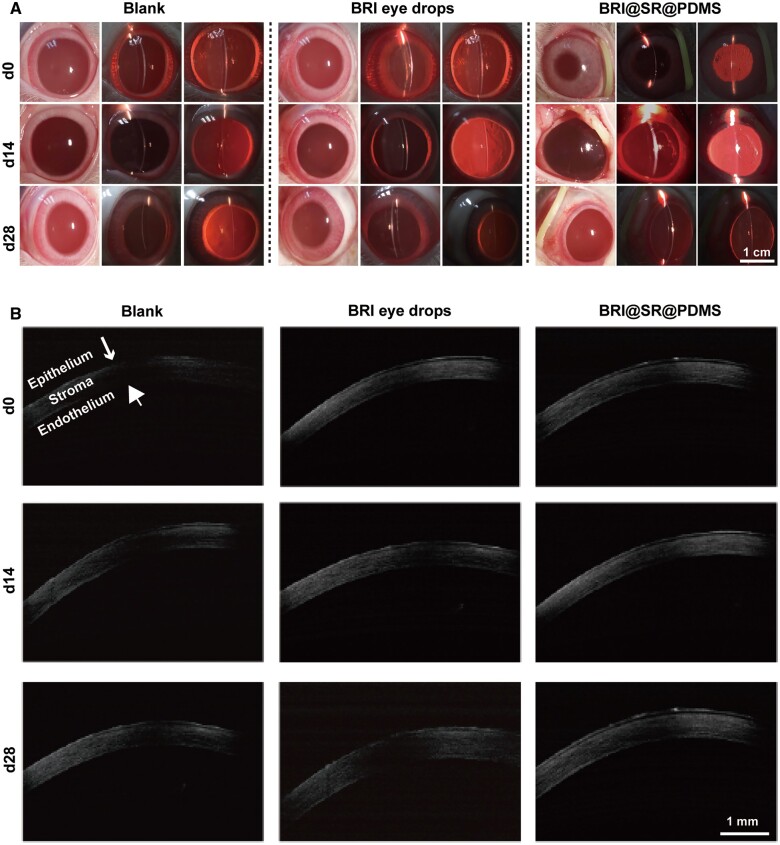
Representative photographs of rabbit ocular anterior segments taken by a slit-lamp with a Canon 600D digital camera (A) and OCT (B) after instillation of BRI eye drops (0.15%) or administration of BRI@SR@PDMS implant.

The *in vivo* biosafety of the BRI@SR@PDMS implant was evaluated via slit-lamp observation and OCT examination of rabbit ocular anterior segments, histopathological analysis of eyeballs and the detection of inflammatory cytokines in the aqueous humor at the observation terminal (Day 28). As shown in Fig. 6A, no obvious signs of inflammation and infiltration were observed at Days 14 and 28 in the anterior segments of rabbit eyes in all groups. Moreover, no obvious corneal thickening and edema were observed in all groups (Fig. 6B). Based upon the above clinical evaluations, the BRI@SR@PDMS implant administrated in the rabbit conjunctival sac had proven to be biocompatible and safe.

**Figure 7.**
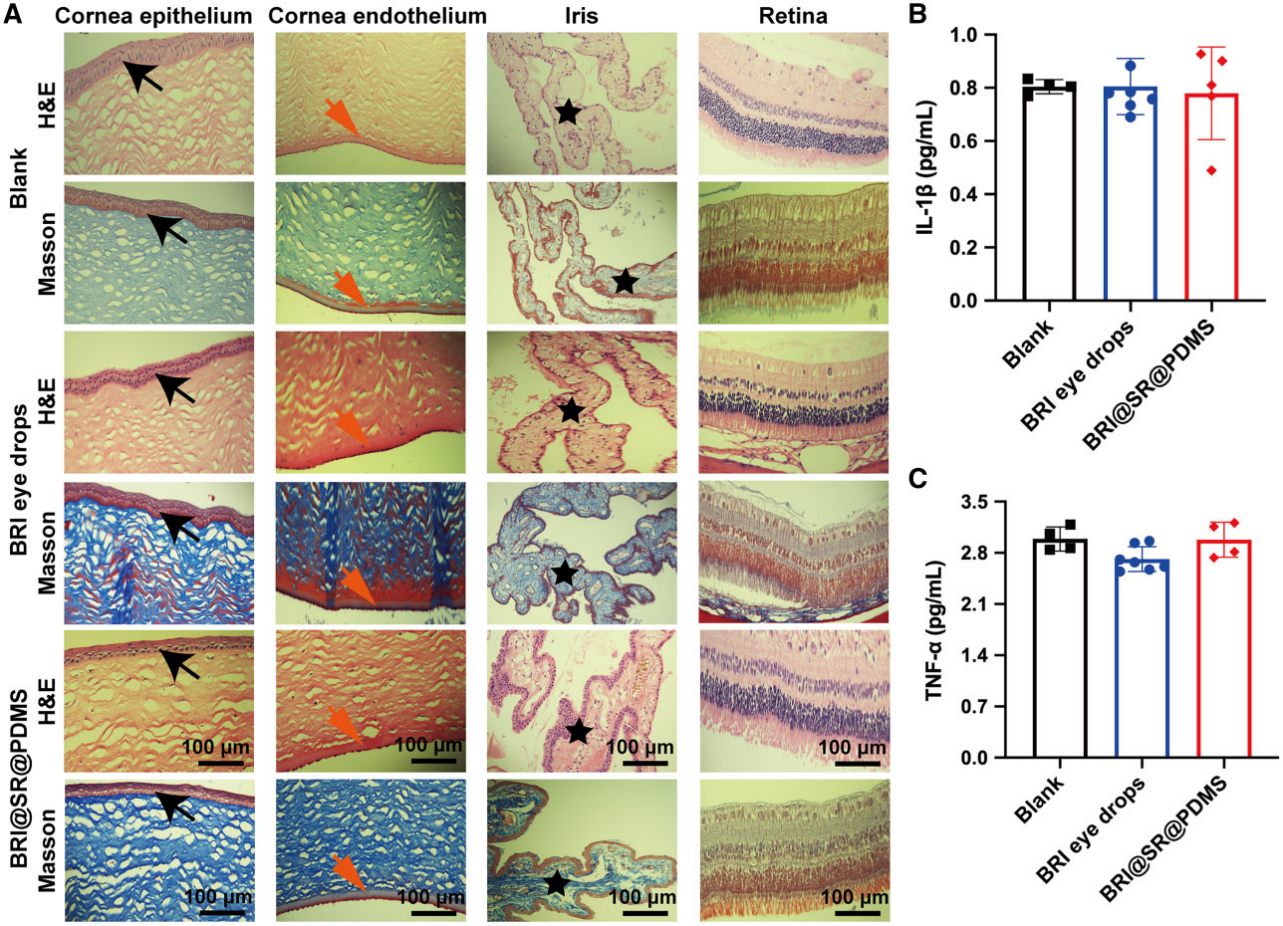
(A) Representative photographs of histological sections of rabbit eyeballs stained by H&E and Masson after instillation of BRI eye drops or administration of BRI@SR@PDMS implant. Epithelium (black arrows), endothelium (red arrows) and iris (five-pointed star). (B) IL-1β and (C) TNF-α concentrations in the aqueous humor at the observation terminal (Day 28) in the BRI eye drops (0.15%) and BRI@SR@PDMS implant groups (*n* = 4), compared with those in the blank group (*n* = 8). *P <* *0.05.

Histological sections of rabbit eyeballs at the observation terminal (Day 28) in all groups were investigated and the representative photographs are presented in Fig. 7A. For all groups, the cornea (from epithelium to endothelium), iris as well as retina were all fully examined. The cornea cross-sections treated with BRI eye drops or BRI@SR@PDMS implant did not show any histological abnormality, such as stromal hyperplasia and keratitis, compared with those in the blank group. The anterior elastic layer, stromal layer and endothelial layer were clear, and the corneal integrity after BRI@SR@PDMS administration was almost unaffected. Besides, there was no infusion of inflammatory cells in the ciliary body and retina. It was therefore concluded that the BRI@SR@PDMS implant possessed good biocompatibility and was safe as an ocular drug delivery system for future anti-glaucoma therapy. Furthermore, the *in vivo* inflammatory cytokines were detected, and the results revealed that the IL-1β and TNF-α levels were at the same level in both BRI@SR@PDMS implant and BRI eye drops, compared with those in the control group (*P *>* *0.05). Hence, all observations indicated that BRI@SR@PDMS implant was non-toxic to rabbit intraocular tissues.

## Conclusion

In this study, a BRI-loaded SR implant coated with PDMS (BRI@SR@PDMS) is successfully developed for the treatment of IOP reduction in glaucoma. The *in vitro* BRI release from BRI@SR@PDMS implant reveals a more sustainable trend lasting over 1 month. The BRI@SR@PDMS implant shows no cytotoxicity to human corneal epithelial cells and mice corneal epithelial cells *in vitro*, and good biosafety *in vivo*. After administrated into rabbit conjunctival sac, the BRI@SR@PDMS implant releases BRI in a sustained fashion and effectively lowers IOP for 18 days, compared with the conventional BRI eye drops with hypotensive effect only for 6 h. Therefore, BRI@SR@PDMS implant is a kind of promising non-invasive platform for long-term reduction of IOP in patients with ocular hypertension or glaucoma.
